# Professional nurses’ lived experiences regarding the performance management system in the Mopani district

**DOI:** 10.4102/curationis.v40i1.1631

**Published:** 2017-10-13

**Authors:** Sheillah H. Mboweni, Lufuno Makhado

**Affiliations:** 1 Department of Health, North West Province, South Africa; 2 Department of Nursing Sciences, North-West University, South Africa

## Abstract

**Background:**

The purpose of the performance management system (PMS) is planning, monitoring, evaluation and development of employees to meet the organisational goals and objectives and transform of service delivery to excellence of the organisation. However, public services are deteriorating and implementation of PMS stimulated different views among professional nurses (PNs) in the Mopani district clinics, which warrants exploration and documentation of the findings.

**Objective:**

The objective of this study was to explore and describe the PNs’ perceptions towards PMS.

**Methods:**

A qualitative, phenomenological research was conducted to explore and describe the nurses’ lived experiences regarding PMS. A purposive sampling method was used. Data collection was done using focus group interviews. Three focus group discussion sessions were conducted, inclusive of six to nine participants in each session.

**Results:**

Twenty-two PNs were interviewed. The findings revealed the following themes: PNs’ uncertainty regarding the implementation of PMS, poor implementation of PMS and its process, lack of knowledge and understanding of PMS implementation and its process, and negative attitudes towards the implementation of PMS.

**Conclusion:**

PNs perceived PMS negatively. There is a need to improve leadership and management behaviour by enhancing productivity, job satisfaction and organisational commitment. Constructive feedback, training and capacity development, including standardisation and stabilisation of performance instrument, might improve the process.

## Introduction and background

The objective of the performance management system (PMS) is to develop employee’s skills and competencies in order to meet the organisational goals and objectives and to transform service delivery (Public Service Regulations 2001). However, the implementation process frustrates and demoralises professional nurses (PNs) in the Primary Healthcare (PHC) clinics against better performance. Performance management processes will help management to identify gaps in knowledge, skills and competency levels of employees and to establish an action plan to develop and capacitate employees. The PMS should be linked with the strategic plan, namely the Annual Performance Plan and PNs’ work plans, in order to ensure synergy. Therefore, this study sought to explore and describe the PNs’ lived experiences regarding PMS.

The PMS is a very important tool in driving the organisation towards the attainment of set goals through quarterly and annual performance appraisals, monitoring and evaluating employees’ performance by rewarding good performance and addressing poor performance. The PMS is adopted globally as it is crucial in the improvement of quality healthcare and employee motivation. Nevertheless, nurses and other healthcare workers globally are dissatisfied and demoralised by the implementation of the PMS both in the public and private sector, resulting in high employee turnover, absenteeism, lack of commitment and enthusiasm, leading to poor quality care of clients, patients and the community (Keegal 2013).

It was further emphasised that PNs are often disappointed by the process of performance appraisal, although there is confidence in its potential value of performance (Vasset, Marnburg & Furunes 2010). Kampkötter (2016) also emphasised that employees have expectations about their individual contributions to the success of the organisation they work for and employees who actually perform well may feel disappointed when they are not adequately rewarded for their achievements with pay raises, bonuses or promotions. The majority of employees often view appraisals as a process to secure salary increases because it serves as a motivation to work harder, thus improving service delivery (Burton 2012). It has been reported that it is difficult for supervisors and managers to measure the performance of nurses because of the nature of the work, changing managers and a lack of knowledge, experience on the processes and the ability to identify the strengths and weaknesses of their supervisees’ performance (Nikpeyma et al. 2014).

A study conducted in Tanzania revealed that PHC workers were neither motivated nor satisfied (Lutwama et al. 2013). Supervisors received negative feedback regarding performance instead of developing an action plan to address the gaps and weaknesses. In South Africa, the PMS was introduced to the public sector in order to improve service delivery, bureaucratic system processes and attitudes and to redress the imbalances of the past (Armstrong 2012; Bach & Edwards 2015). However, there are still challenges regarding PMS in the PHC setting, which promotes poor service delivery.

### Problem statement

PMS has been introduced among nurses to improve the performance and quality in the delivery of healthcare services. However, the services in the PHC clinics and Community Health Centres (CHCs) are deteriorating gradually. Managers are expected to conduct performance appraisal among PNs according to the PMS policy. Unfortunately, the implementation of the PMS policy and the process has been seen to frustrate and demoralise nurses. Managers and PNs seem to have some misunderstanding regarding the goals and objectives of the PMS. There is little or no evidence of PNs’ experiences regarding PMS in the Limpopo Province setting. Hence, a study was conducted to explore the PNs’ lived experiences regarding the PMS in Mopani district clinics, Limpopo Province.

### Aim of the study

The purpose of the study was to explore and describe the lived experiences of PNs regarding PMS and to recommend an improved implementation process. The study aimed to answer the following question: What are the lived experiences of PNs regarding the PMS implementation?

### Research objectives

The objective of the study was to explore and describe the lived experiences of PNs regarding the PMS implementation.

### Definition of key concepts

*Performance* is the act, process and art of functioning or conduct (Collins Concise Dictionary n.d.).

*Performance appraisal* is the assessment or evaluation of quality of work performed by an employee.

*Performance management system* is a policy framework and procedure document that guides the organisation‘s performance planning, monitoring, evaluation, measurement, review, reporting and improvement as well as defines the roles of different role players. It is a cycle that consists of three phases.

*Professional nurse* refers to a person who has qualified to practise as a professional and has met the requirements as prescribed by the South African Nursing Council (2005). In this study, ‘PN’ refers to PNs and includes Clinical Nurse Practitioners, Clinical Nurse Specialists and managers in the district clinics.

*Primary Healthcare* is essential healthcare based on practical, scientifically sound and socially acceptable methods and technology, which is accessible and affordable to individuals and families in the community through their full participation to achieve self-reliance and self-determination (African National Congress 1994:20).

*Clinic* is a place in which patients or clients are given PHC services, which include medical treatment, education, information, advice and counselling in the community.

### Contribution of the study to Performance Management System

The study contributed to a better understanding of the influences of the implementation of the PMS in the healthcare system, which include the lived experiences, understanding and knowledge of PNs regarding the PMS and its processes. This provides the basis for improvement in the delivery of healthcare services in the public services through the implementation of recommendations made.

## Research methods

### Research design

A qualitative, phenomenological research design was used in the study, as it provided an in-depth, rich and complex description of the lived experiences of PNs regarding the PMS in one of the districts’ clinics of Limpopo Province (Grove, Burns & Gray 2013).

### Context of the study

The study was conducted at two PHC and one CHC facility around Mopani district, Limpopo Province. These facilities were chosen based on their centrality and availability of a boardroom for privacy. Furthermore, the centrality of the chosen facilities was facilitated by the number of PNs who agreed to participate in the study. Mopani district is predominantly rural. The districts consist of 95 PHC clinics and 8 CHCs (Limpopo Department of Health 2016).

### Population and sampling

The population of the study included all PNs working in the PHC and CHC facilities around Mopani district who had undergone performance appraisal. Purposive sampling was used to select PNs who were knowledgeable and had experienced the PMS process. All nurses who had undergone performance appraisal from all PHC and CHC facilities were recruited for the study. The sample incorporated six to nine PNs per focus group and the study sample size consisted of a total of 22 PNs after data saturation was reached.

### Data collection method

Three focus group discussions or interviews (FGD) were conducted with 22 PNs, 1 male and 21 female. Data saturation, which emphasises that data should be collected until there are few surprises in the data and no more patterns or themes are emerging from the data (O’Reilly & Parker 2012), was achieved with this sample. A FGD allowed the researcher to gain a better understanding of PNs’ lived experiences regarding the implementation of the PMS. The FGD was conducted in a boardroom of each of the three central clinics chosen to allow participants not to travel long distances, with a closed door to promote privacy and to discourage interruptions. The researchers were given permission from the clinic management to utilise the boardroom. Both English and Xitsonga were used during the FGD to allow every participant to provide a rich description of his or her experience. The participants and researchers were Xitsonga-speaking people and fluent in the language. The discussions were recorded after permission was obtained from participating PNs.

All PNs were requested to complete their demographic data, which also served as an icebreaker before the actual FGD. Both researchers were experienced in the facilitation of FGD. Non-verbal behaviour, feelings, responses and mannerisms were noted in the field notes. A semi-structured interview guide was used to guide the FGD. The interview guide was inclusive of probes that were applicable to the FGD about specific experiences provided by the PNs, such as: ‘tell me more about that’, ‘what do you mean by that?’, ‘what else can you tell me?’ and ‘is there anything else that you would like to say?’. These allowed PNs to expand on the specific experiences they reported. The first section obtained participants’ demographic data and the second section focused on experiences regarding the PMS. The interview schedule was piloted with six PNs from Greater Letaba clinics of Mopani district and changes were made with regard to the phrasing of the questions.

### Data analysis

Phenomenological steps (Table 1) were used to organise, manage and analyse data as well as to guide the study (Streubert-Speziale & Carpenter 2011). Microsoft Excel was used to analyse demographic data and descriptive statistics were used to summarise them. Literature was reviewed in relation to themes that emerged.

**TABLE 1:** Five phenomenological steps for qualitative analysis.

Step	Description of data analysis activities
Descriptive phenomenology	Intuiting: conducting focus group discussion and managing data. Analysis: coding and categorising. Describing: identifying main ideas that emerge from coded data.
Phenomenology of essence	Involves reduction of data by linking related ideas to discover meaning.
Phenomenology of appearances	Involves data synthesis by identifying and describing main themes.
Constitutive phenomenology	Arriving at conclusions and recommendations based on McNeese-Smith modelling theory (McNeese-Smith, 1996).
Reductive phenomenology	Done throughout the steps and study by bracketing all preconceived ideas, personal assumptions and bias.

*Source*: Authors’ own work

### Trustworthiness

Trustworthiness involves ensuring credibility, dependability, conformability and transferability (Polit & Beck 2008). Trustworthiness with regard to data collection was enhanced by conducting a pilot study, which served as a pre-test to the interview schedule and interviewing skills, with PNs from six clinics in the Greater Letaba Municipality; this was excluded from the study. The researcher spent enough time with each group, being 90–120 minutes. Recorded interviews, transcribed data and interpretations were shared with participants in order to confirm or validate if their experience was actually captured. Participants were supplied with the researcher’s personal and academic information. An audit trail was maintained by keeping all copies of notes and transcribing and recording data for future use. An independent co-coder was used and three consecutive discussions were followed before consensus was reached.

### Ethical considerations

Permission to conduct the study was given by the University of South Africa Research & Ethics Committee, Limpopo Department of Health and Social Development (Ref no. 4/2/2) and Mopani district Primary Heathcare (Ref no. S5/4/1). All participants volunteered their time to participate in the study.

## Results

### Demographic characteristics

The majority of PNs were female (95%; *n* = 21) with 10–13 years’ experience, working as Clinical Nurse Practitioners (36.3%; *n* = 8). The PNs’ ages ranged from 31 years and above and the majority were 40–45 years old (63.6%; *n* = 14). It was evident that most PNs had a Postgraduate Diploma (36.3%; *n* = 8) but they never received training on PMS in clinical, education or health service management.

### Professional nurses’ lived experiences regarding the implementation of Performance Management System

The study findings revealed three (3) themes, namely PNs’ uncertainty regarding the implementation of PMS, knowledge and lack of understanding of PMS and negative attitude of PNs towards PMS (see Table 2).

**TABLE 2:** Lived experiences of professional nurses regarding performance management system implementation.

Theme	Subtheme	Categories
PNs’ uncertainty regarding the implementation of PMS	Poor implementation of PMS and its process	Bias and subjectivity
		Lack of feedback
		Lack continuous monitoring and evaluation of performance
	Emotions towards PMS	Fear
		Frustrations
		Demoralisation
	Inadequate resources	Lack of material resources
		Shortage of human resources
	Unclear policies on the implementation of PMS	PMS is financially orientated
		Different instruments utilised
Knowledge and understanding of PMS	Lack of knowledge and understanding of PMS	PNs’ lack of knowledge and understanding of PMS
		Managers’ lack of knowledge and understanding of PMS
	Lack of capacity building	Lack of training among managers on PMS
Attitudes of PNs towards PMS implementation	Negative attitudes towards PMS	Loss of interest towards PMS
		Favouritism in PMS
		Poor interpersonal relationship between staff and managers
Consequences of poor performance appraisal	Performance appraisal negatively influences service delivery	Poor service delivery because of PMS

*Source*: Authors’ own work

PMS, Performance Management System; PN, professional nurse.

### Theme 1: Professional nurses’ uncertainty regarding the implementation of Performance Management System

The uncertainty regarding the implementation of PMS was evident through poor implementation of PMS and its process, emotions towards PMS, inadequate resources and unclear policies on the implementation of PMS.

#### Subtheme 1: Poor implementation of Performance Management System and its process

This subtheme was inclusive of bias and subjectivity, lack of feedback and no continuous monitoring and evaluation of PNs’ performance in the implementation of PMS and its processes.

**Bias and subjectivity in the performance management system implementation and its process:** PNs perceived PMS as biased and reacted with anger towards its process and implementation. The PMS had been reported to lack fairness, openness and transparency. This was supported by the following direct expressions from PNs:

‘PMS lacks openness and transparency; there is favouritism and nepotism in rating performance, it discourages me. [*Emotional*] Unexceptional attachments as evidence are demanded while others don’t even attach anything.’ (P2, FGD 2, Female)

Participants also perceived the PMS as being subjective. There were no clear guidelines about how PMS was implemented. Rating during PMS appraisal solely depended on the supervisor or manager, especially with the rating of competencies. One of the PNs remarked:

‘Managers have different perceptions on performance appraisal and argues a lot in the PMS committee [*lashing hands and emotional*].’ (P6, FGD 1, Female)

**Lack of feedback:** PNs reported that they did not receive regular feedback for their quarterly and final annual performance. This prevented them from acquiring knowledge about their strengths and weakness regarding their key performance areas and further deprived them from improving where they lacked. PNs revealed that:

‘We do not receive feedback on the ratings or development plan to improve performance and I just receive performance bonus without knowing my weakness and strength with regard to my performance.’ (P4, FGD 3, Female)

**Lack of continuous monitoring of performance**: It was evident from PNs as they further indicated that monitoring of performance in the form of quarterly performance appraisal was not done. PNs only submitted all documents for all quarters at the end of the financial year. Hence, there was no continuous appraisal and feedback regarding PMS. This was reiterated by a PN stating that:

‘There is no on-going or periodic and final feedback.’ (P2, FGD 3, Female)

#### Subtheme 2: Emotions towards Performance Management System

This subtheme incorporated fear, frustration and demoralisation as experienced by PNs.

**Fear:** PNs revealed that PNs who were in a management position or supervisors were afraid to rate an employee one or two (unsatisfactory) because they were being threatened. A PN’s statements confirmed this:

‘I am afraid to approach a person because of his/her poor performance; some subordinates are cheeky, hostile, intimidating and threatening with witchcraft [*with a sad voice*].’ (P3, FGD 2, Female)

**Frustrations:** It was also indicated that the implementation of PMS and its process frustrated PNs in such a way that they ended up feeling dissatisfied to work in such an environment. This was true as PNs were expected to provide inconspicuous documents on demand as evidence of duties performed given the interrelatedness of the nursing duties. PNs verbalised that

‘Unexceptional attachments as evidence are demanded, very difficult as some nursing duties are interrelated.’ (P7, FGD 3, Female)

**Professional nurses feel demoralised:** Most PNs reported that they felt demoralised about the process and implementation of PMS and felt less enthusiastic towards performing at their best to improve their performance.

‘The PMS frustrates and demoralises me, and I no longer have the zeal to perform.’ (P1, FGD 3, Female)

#### Subtheme 3: Inadequate resources towards the implementation of Performance Management System

PNs reported that there was an inadequate supply of relevant resources necessary for effective completion of the documents needed for performance management agreement and appraisal, which were also found to be changing every time, thus lack of material resources and shortage of human resources.

**Lack of material resource:** There were no photocopy machines and stationery to assist PNs to produce and complete the necessary information and this lack of material resources delayed the submission process and each PN was responsible for completing and submitting his or her own PMS form. Furthermore, this resulted in PNs resorting to using their own funds which was reported to promote stress among PNs. This was evident through the statement given by a PN:

‘I experience problems with lack of photocopy machine, stationery as forms always changes, it is financially stressing.’ (P3, FGD 1, Female)

**Shortage of human resource**: PNs confirmed that there was severe if not high shortage of staff and some managers were failing to conduct quarterly performance appraisal as they had to perform clinical work to curb the shortage. One PN verbalised that:

‘Shortage of staff increases workload and delays the completion of PMS reports.’ (P3, FGD 1, Female)

#### Subtheme 4: Unclear policies in the implementation of Performance Management System

The study findings revealed that there were unclear policies regarding the process and implementation of PMS; hence, PNs reported that PMS was a financially orientated system and there were different instruments utilised for PMS.

**Financially orientated system:** The PMS was not serving its objective of developing PNs but was seen as a routine practice that was done yearly for the purpose of getting or providing incentives in the form of bonuses. Both good and poor performers received equal incentives. The PMS had been said to focus only on money which was not even enough. This was verbalised as follows:

‘PMS is an ATM where employees withdraw money and it is not enough we all receive flat rate.’ (P4, FGD 2, Male)

**Use of different performance management system instruments**: PNs indicated that there were untimed changes in the format of the performance agreement or appraisal instrument. PNs in the district clinics used different forms. The forms were always sent back for corrections and that frustrated PNs. One PN reiterated that:

‘Forms changes every day, I always run around looking for the forms and asking how to complete it.’ (P2, FGD 2, Female)

### Theme 2: Knowledge and understanding of Performance Management System

The theme consisted of two subthemes, namely lack of knowledge and understanding of PMS and lack of capacity building regarding PMS.

#### Subtheme 1: Lack of knowledge and understanding of Performance Management System

Lack of knowledge and understanding of PMS was evidently reported to be present in both PNs and managers or supervisors.

**Professional nurses’ lack of knowledge and understanding of performance management system:** It was evident and reported by PNs that they had inadequate knowledge when it came to the proper process and how PMS should be implemented. A lack of understanding of the process confused and frustrated PNs as they could not understand the process thereof. This was verbalised by a PN as follows:

‘We are not provided with adequate information about what needs to happen during performance agreement and most of issues are just said but not explained which frustrates and confuses us because we do not understand the purpose of this performance system.’ (P4, FGD 2, Male)

**Managers lack knowledge and understanding of the purpose the performance management system:** Participants reported that managers or supervisors in the clinics lacked knowledge and understanding of the purpose of PMS as per policy. They did not have the policy in the clinic.

‘When I ask my manager to assist, she indicated that she is not clear on how to do it, and has never seen the policy but only told by HR office that it is available somewhere.’ (P5, FGD 1, Female)

#### Subtheme 2: Lack of capacity building

Lack of capacity building was revealed to be inclusive of lack of training among managers or supervisors on PMS.

**Lack of training among managers on performance management system:** PNs indicated that they did not receive any formal training on the process or implementation of PMS or in-service training with regard to any changes related to the PMS process. PNs were recorded as saying:

‘I am a manager but I did not receive training on PMS but had been orientated by others; I have never attended any workshop or in-service training.’ (P1, FGD 2, Female)

### Theme 3: Attitude towards the Performance Management System

This theme incorporated a negative attitude towards PMS.

#### Subtheme 1: Negative attitude towards the Performance Management System

Negative attitudes were revealed through a loss of interest towards PMS, favouritism in PMS and poor interpersonal relationships between staff and managers.

**Loss of interest towards Performance Management System**: PNs reported losing interest and having developed a negative attitude towards the system because of bias, subjectivity and favouritism in the rating of performance. A PN confirmed that:

‘I just contract [*To PMS*] due to fear of disciplinary hearing, I have negative attitude towards it because I do not have adequate knowledge, I think this PMS should be discarded it’s not serving the purpose.’ (P3, FGD 2, Female)

**Favouritism in Performance Management and Development System**: One participant’s expression confirmed favouritism in terms of performance appraisal and resulted in poor relationships among staff and managers:

‘PMS lacks openness and transparency, and there is favouritism and nepotism in rating performance, it discourages me [*Emotional*].’ (P5, FGD 1, Female)

**Poor interpersonal relationship between staff and managers**: One participant reported that unfair ratings created tension among staff members and managers resulting in poor communication and distorted teamwork:

‘It’s unfair, how somebody who’s not performing well, always not on duty can get to be rated more and better than myself [*Emotional*].’ (P4, FGD 2, Male)

### Theme 4: Consequences of poor performance appraisal

Poor performance appraisal was reported to bear consequences and this theme was composed of one subtheme, thus performance appraisal negatively influences service delivery.

#### Subtheme 1: Performance appraisal negatively influences service delivery

This subtheme consisted of and described poor service delivery because of PMS.

**Poor service delivery because of performance management system**: Participants reported with concern that poor implementation of PMS had a negative influence on the quality of service delivered and health outcomes. This also promoted lack of enthusiastic approach towards service delivery as PNs lacked motivation to carry on. This can explain the fact that the PHC and CHC facilities were not achieving the set target for the District Health System. One PN verbalised that:

‘I don’t care even if we don’t achieve the set target, or when clients are not satisfied, I’m demoralise about this PMS.’ (P3, FGD 2, Female)

## Discussion

### Professional Nurses’ uncertainty regarding the implementation of Performance Management System

The PNs in the district clinics perceived the PMS negatively and emotionally because there was bias in the implementation of the process by managers and supervisors. This perception needs to be corrected as it frustrates and demoralises the nurses against better performance. In a similar study on employees’ perceptions on performance appraisals, Reb and Greguras (2015) reported that there was inconsistency and bias in the implementation of PMS and it weakened the performance appraisal. A lack of clear guidelines on how to implement PMS in the PHC clinics by PNs exposed PMS to managers’ or supervisors’ subjectivity rather than objectivity. This made performance appraisal biased and resulted in PMS being viewed as an ineffective approach towards performance management. PNs also revealed that they were not evaluated according to their performance.

Money was seen as the only reward that could be offered in PMS among PNs. Furthermore, Porter (2004) emphasised that focusing on money and profit can be a fruitless effort when it comes to PMS among PNs. The organisations need to aim at developing employees in order to achieve their goals and objectives. Managers need training to understand processes and to reduce bias (McWalter, Alkhenizan & Hussain 2014). However, the importance of intrinsic incentives should be emphasised among all managers and proper validation of performance instruments should be done and monitored on a yearly basis.

The performance appraisal and rating were deemed unfair by PNs. Negative rating might lead to low productivity and turnover as well as absenteeism (Mokgolo, Mokgolo & Modiba 2012). A similar study conducted by Ramulumisi, Schultz and Jordaan (2015) revealed that there was no fairness in performance appraisal, nor any satisfaction about the process. Nepotism and favouritism in performance appraisal led to unfair PMS and confirmed the extreme lack of understanding of the objectives of the PMS by managers and supervisors. PNs became demotivated and demoralised by a lack of fairness and this might have had a negative impact on performance, quality of care rendered (Manyaka & Sebola 2012) and eventually poor health outcomes in the public sector.

Managers and supervisors are fearful in managing PNs who perform poorly in the PHC clinics. Atefi et al. (2014) declared that managers lack strategies on the management of poor performers and always give an impression that it is not diplomatic to address poor performance in order to avoid dialogue in confronting them. Managers lack skills and strategies to deal with poor performers and this has a serious impact on the performance, growth and development of PNs.

Enabling resources to implement the system are lacking in the PHC clinics. Bakker et al. (2016) indicated that a lack of resources, be it material or human, in combination with excessive job demands, has a negative influence on performance and might lead to stress and burnout. PNs need resources to perform their allocated tasks. A lack of such resources frustrates them and may delay submission or lead to no submission of performance appraisal as per guidelines. This affects quarterly monitoring of progress in achieving set targets, identification of gaps and the development of PNs including annual evaluation of PNs’ performance. PNs cannot provide evidence of the work done as they lack material to record services rendered to the clients.

Nurses receive negative, demoralising feedback and rewards. Employees receiving negative, unfavourable performance outcomes tend to have negative attitudes towards their jobs and do not perform well (Bakker et al. 2016).

Managers lack skills and understanding of the importance of positive constructive criticism in performance appraisal rather than negative feedback, to harness potential and improve performance of PNs. Managers lack training on how to improve employees’ performance after identifying weakness. This is in line with a study conducted in South Africa by Brand and Pretorius (2003), stating that there was a lack of employees’ development, training and coaching, resulting in poor performance. Conversely, Boland and Fowler (2000) stated that there had been a major development in performance measurement since the introduction of the PMS by the National Health System in South Africa. Managers lack formal and informal PMS training to empower them so that they can effectively and efficiently implement the PMS among PNs, thus motivate them to transform the public health service and instil the culture of excellent performance.

Untimely changes in performance instruments frustrate PNs. Reb and Greguras (2015) explained that changes in performance instruments may influence rating and needs consultation between the one conducting the rating and the one being rated. Frequent unplanned changes about the performance instrument without formal communication and in-service training among PNs and other stakeholders involved in PMS have a potential to cause confusion in the implementation thereof.

Professional nurses have demonstrated negative attitudes towards the implementation of PMS. Favouritism during performance appraisal has created tension, poor communication and interpersonal relationships between PNs and managers and even among PNs themselves. Favouritism destroys teamwork among PNs. This is supported by Fu (2015) revealing that favouritism creates ethical dilemmas and, if not dealt with appropriately, it has the potential to demoralise staff and affect performance negatively. Smith et al. (2015) and Du et al. (2012) also indicated that favouritism and bias in performance ratings create tension among employees of different races, ages, genders, political affiliations and positions in the working environment and affect achievements of the organisational goals and objectives. The service delivery in the district clinics is poor or deteriorating as the PNs are demoralised and frustrated about the PMS. This situation will lead to non-achievement of the Department’s strategic goals and objectives, including the objectives of the PMS in the public sector.

### Practical implications

Based on the results of the perceptions and attitudes of PNs with regard to PMS, the recommendations expressed by PNs might practically improve the implementation of the system and harness the performance and quality of healthcare services in the public sector. Further research can be conducted:

- to explore other performance management frameworks that can be used to evaluate nurses as they provide interrelated roles
- to evaluate the effectiveness of the PMS among nurses.

Nursing education should include PMS in the nursing management curriculum, so that they can assess employees properly. Skills development and training should prioritise training PNs on PMS.

### Limitations of the study

Only PNs working in the Greater Giyani Municipality PHC clinics of Mopani district were part of the study. The results cannot be generalised beyond the area. Despite these limitations, the research findings have essential implications.

### Recommendations

Based on the results of the study, the following can be recommended guided by McNeese-Smith (1996):

#### Standardisation and stabilisation of performance instruments

The Provincial Department of Health Human Resources should develop the performance instrument that can be used by all nurses in the province. Changes should be effected periodically at the end of the performance cycle after consulting all relevant stakeholders and conducting in-service training for all before implementation.

#### Training and capacity development to address identified gaps

A Training and Development plan is necessary to close the identified gaps during appraisal and to also entail proper budgeting by the Human Resource Development unit. Managers and supervisors can apply an Integrated Performance Management and Development System to improve and instil individual commitment towards improved performance and service delivery.

#### Communicating performance results or feedback constructively

After moderation of the performance appraisal, managers or supervisors should provide constructive feedback to employees of the outcomes, either good or poor performance, in writing or verbally.

**Policies on performance management that promote equality:** Policies should not only be in place but be implemented to promote equality and fairness during presentation and moderation of performance assessments. The presence of labour relations or union representative officials is necessary. This also reduces complaints, dissatisfaction and strikes. Human resources should ensure that all facilities comply with the involvement of such stakeholders in all their assessment.

#### Accessibility of resources to implement the Performance Management System

The Department of Health should ensure that there are enough resources for PNs to perform their jobs. This includes material, equipment, staff, infrastructure and finance. Resources enable PNs to perform better and to improve the quality of health services.

**Continuous monitoring and evaluation of performance:** Managers should ensure that continuous quarterly monitoring is done to track performance, identify gaps and provide remedy so that there is fair final evaluation at the end of the performance cycle.

**Introduction of various methods of incentives:** The purpose and objectives of the PMS should always be taken into consideration when deciding on the incentives. Both extrinsic and intrinsic incentives are necessary to motivate PNs to perform better. Caution should be taken not to emphasise money by over-appreciating verbally or in the form of a certificate or trophy for employees who perform well.

## Conclusion

The study revealed that PNs perceived PMS negatively and this hindered the achievement of the Department’s goals and objectives. It was perceived as more financially oriented ignoring other forms of rewards. The lack of knowledge and training among PNs led to these negative perceptions. This hindered the quality performance and development. Intervention should be based on recommendations expressed by nurses.
